# Socioeconomic status and treatment outcomes for individuals with HIV on antiretroviral treatment in the UK: cross-sectional and longitudinal analyses

**DOI:** 10.1016/S2468-2667(16)30002-0

**Published:** 2016-11

**Authors:** Lisa S Burch, Colette J Smith, Jane Anderson, Lorraine Sherr, Alison J Rodger, Rebecca O'Connell, Anna-Maria Geretti, Richard Gilson, Martin Fisher, Jonathan Elford, Martin Jones, Simon Collins, Yusef Azad, Andrew N Phillips, Andrew Speakman, Margaret A Johnson, Fiona C Lampe

**Affiliations:** a Research Department of Infection and Population Health, University College London, London, UK; b Centre for the Study of Sexual Health and HIV, Homerton University Hospital NHS Foundation Trust, London, UK; c Barts Health NHS Trust, London, UK; d Institute of Infection and Global Health, University of Liverpool, Liverpool, UK; e Royal Sussex County Hospital, Brighton, UK; f School of Health Sciences, City, University of London, London, UK; g East Sussex Healthcare NHS Trust, Eastbourne, UK; h HIV i-Base, London, UK; i National AIDS Trust, London, UK; j Royal Free London NHS Foundation Trust, London, UK

## Abstract

**Background:**

Few studies have assessed the effect of socioeconomic status on HIV treatment outcomes in settings with universal access to health care. Here we aimed to investigate the association of socioeconomic factors with antiretroviral therapy (ART) non-adherence, virological non-suppression, and virological rebound, in HIV-positive people on ART in the UK.

**Methods:**

We used data from the Antiretrovirals, Sexual Transmission Risk and Attitudes (ASTRA) questionnaire study, which recruited participants aged 18 years or older with HIV from eight HIV outpatient clinics in the UK between Feb 1, 2011, and Dec 31, 2012. Participants self-completed a confidential questionnaire on sociodemographic, health, and lifestyle issues. In participants on ART, we assessed associations of financial hardship, employment, housing, and education with: self-reported ART non-adherence at the time of the questionnaire; virological non-suppression (viral load >50 copies per mL) at the time of questionnaire in those who started ART at least 6 months ago (cross-sectional analysis); and subsequent virological rebound (viral load >200 copies per mL) in those with initial viral load of 50 copies per mL or lower (longitudinal analysis).

**Findings:**

Of the 3258 people who completed the questionnaire, 2771 (85%) reported being on ART at the time of the questionnaire, and 2704 with complete data were included. 873 (32%) of 2704 participants reported non-adherence to ART and 219 (9%) of 2405 had virological non-suppression in cross-sectional analysis. Each of the four measures of lower socioeconomic status was strongly associated with non-adherence to ART, and with virological non-suppression (prevalence ratios [PR] adjusted for gender/sexual orientation, age, and ethnic origin: greatest financial hardship *vs* none 2·4, 95% CI 1·6–3·4; non-employment 2·0, 1·5–2·6; unstable housing *vs* homeowner 3·0, 1·9–4·6; non-university education 1·6, 1·2–2·2). 139 (8%) of 1740 individuals had subsequent virological rebound (rate=3·6/100 person-years). Low socioeconomic status was predictive of longitudinal rebound risk (adjusted hazard ratio [HR] for greatest financial hardship *vs* none 2·3, 95% CI 1·4–3·9; non-employment 3·0, 2·1–4·2; unstable housing *vs* homeowner 3·3, 1·8–6·1; non-university education 1·6, 1·1–2·3).

**Interpretation:**

Socioeconomic disadvantage was strongly associated with poorer HIV treatment outcomes in this setting with universal health care. Adherence interventions and increased social support for those most at risk should be considered.

**Funding:**

National Institute for Health Research.

## Introduction

Substantial evidence exists of socioeconomic inequalities in the prognosis of chronic diseases. In Europe and the USA, socioeconomic factors such as poverty, low income, and low education level have been associated with poorer outcomes for several diseases, including cancer, and cardiovascular disease.^1^, ^2^, ^3^ Findings of other studies have suggested that lower socioeconomic status (measured by education or income) is associated with poorer adherence to treatment, such as steroids for asthma^4^ and insulin for diabetes.^5^

HIV is a disease that disproportionately affects those with socioeconomic disadvantage.^6^ In the USA, in people with HIV receiving antiretroviral therapy (ART), lower levels of socioeconomic status (as indicated by lower education level, unemployment, homelessness, or household poverty) are associated with having poorer virological and immunological outcomes.^7^, ^8^, ^9^, ^10^, ^11^ HIV-positive populations in the UK and Europe also comprise distinct demographic groups, with substantial variation in social circumstances. As such, social inequalities may result in disparities in HIV health outcomes. However, in contrast to the USA, the UK has universal free access to health care, including HIV diagnosis, hospital consultations, and antiretroviral treatment, which should greatly lessen financial barriers to accessing HIV treatment and care. Therefore, the associations between socioeconomic factors and HIV outcomes in the USA might not be generalisable to settings with free universal health care, which have been little studied.^11^ Findings of two large European studies, the Swiss HIV Cohort study^12^ and the Spanish CoRIS study,^13^ showed that lower education level was associated with increased odds of viral load being higher than 50 copies per mL at 12 months after ART initiation (unadjusted odds ratios of 1·3 and 1·9, respectively); however, the Danish HIV Cohort study^14^ noted no clear association. Additionally, in the Italian ICoNA cohort study^15^ in individuals who had been taking ART for at least 6 months, unemployment was associated with double the risk of virological failure compared with working full-time. No previous studies have looked at socioeconomic variations in virological outcomes in people treated for HIV in the UK.

**Research in context**

**Evidence before this study**

We searched PubMed for studies assessing the associations between socioeconomic status and HIV treatment outcomes (search originally done in June, 2015, updated in February, 2016, and published in May, 2016). The papers included in the review were original research studies of any design and including secondary observational analyses of randomised controlled trial data in which the criteria for inclusion were: written in English; set in high-income countries; included more than 100 participants; recruitment not entirely before the modern highly active antiretroviral therapy era (ie, some recruitment after 2001); all participants prescribed antiretroviral therapy (ART); not solely reporting analyses adjusted for adherence. We used the following MeSH terms: “HIV” and any of “socioeconomic” or “socio-economic” or “antiretroviral” or “ART” with any “virologic” or “virological” or “immunologic” or “immunological” or “failure” of “rebound” or “suppression” or “viral load” or “CD4” or “education level” or “employment” or “housing” or “occupation” or “deprivation” or “poverty” or “income” or “insurance”. 46 studies met the entry criteria, of which, ten (71%) of 14 noted an association between lower socioeconomic status and poorer virological response, four (67%) of six found an association between lower socioeconomic status and poorer immunological response, and 23 (66%) of 35 found an association between lower socioeconomic status and ART non-adherence. Most studies have been done in the USA (ie, without universal free access to health care) and have focused mainly on education rather than markers of current poverty and hardship. No previous studies have been done of socioeconomic status and ART response in people with HIV in the UK.

**Added value of this study**

Our study provides evidence, from both cross-sectional and longitudinal analyses, that socioeconomic disadvantage (measured by financial hardship, non-employment, unstable housing status, and lower educational level) is an important determinant of HIV treatment outcomes in a setting with universal free access to health care and high rates of treatment success. Thus, our data suggest that the adverse effect of socioeconomic disadvantage goes beyond the ability to access or pay for treatment and care.

**Implications of all the evidence**

Collection of information about socioeconomic factors in a routine clinical care setting is key to identifying individuals at greater risk of poorer virological response to ART. Adherence and social support for socioeconomically disadvantaged individuals should be regarded as an important component of clinical care.

ART non-adherence is the major determinant of virological non-suppression and subsequent virological rebound,^16^ which in turn predicts poorer prognosis for people living with HIV.^17^ Thus, any effect of socioeconomic status on virological outcome is likely to be mediated to a great extent through differential patterns of adherence to HIV treatment. Findings of some European studies^11^, ^18^, ^19^, ^20^ have shown that lower socioeconomic status (measured by education, employment, and social support) is associated with ART non-adherence, but a minority of studies found no evidence.^11^, ^21^

Here, with data from the Antiretrovirals, Sexual Transmission Risk and Attitudes (ASTRA) study, we aimed to investigate the association of socioeconomic factors with ART non-adherence, virological non-suppression, and virological rebound, in HIV-positive people on ART in the UK.

## Methods

### Study design and participants

ASTRA is a cross-sectional, questionnaire study of 3258 HIV-diagnosed individuals in the UK recruited from eight HIV outpatient clinics between Feb 1, 2011, and Dec 31, 2012.^22^ Participants self-completed a confidential questionnaire on sociodemographic, health, and lifestyle issues. The most recent HIV viral load and CD4 count results available at the time of the questionnaire were recorded for all participants by study personnel. Six of the eight study clinics provided linkage to routine HIV clinical records (including serial viral load measurements) for consenting participants (2983 [92%]) using a pseudo-anonymised study number.

Demographic factors, socioeconomic factors, ART use and start date, and ART adherence were self-reported on the questionnaire. The demographic factors of interest were: gender/sexual orientation (men who have sex with men, heterosexual men, women), ethnic origin (white or non-white), and age (as a continuous variable). Men were classified as men who have sex with men if they self-identified as gay or bisexual, or reported sex with a man in the past 3 months. Four markers of socioeconomic status were considered: ability to afford basic needs (financial hardship with four levels); employed (yes or no); housing status (homeowner; renting; unstable or other); and university education (yes or no). The following variables were additional markers of social circumstances: time living in the UK (UK born, >5 years, ≤5 years), English reading ability (UK born, fluent, not fluent), supportive network (most, medium, least), current stable partner (yes or no), and children (yes or no).

Financial hardship was derived from the question “Do you have enough money to cover your basic needs? (Eg, food and heating)” for which responses were: “Yes, all of the time”; “Yes, most of the time”; “Yes, some of the time”; “No”. “Employed” included those who reported either full-time or part-time employment (or self-employment). For housing status, “rented” included those who rented privately or from the council or housing association; “unstable or other” included those living in a hostel, shelter, squat, other temporary accommodation; those staying with partner, family, or friends; and those who were homeless. “Supportive network” aimed to measure supportive relationships based on a modification of the Duke UNC Functional Social Support Questionnaire.^23^ Participants scored from 1: “much less than I would like” to 5: “as much as I would like”, on five items: whether they have people who care what happens to them; they receive love and affection; they get chances to talk to someone they trust; they get invited to do things; they get help when sick. Scores were classified as follows: 5–12 “least support”; 13–24 “medium support”; 25 “most support.”

Ethical approval was obtained via the North West London REC 2 research ethics committee (ref 10/H0720/70).

### Cross-sectional analysis

We assessed the associations of socioeconomic and social circumstance factors with ART non-adherence and virological non-suppression at the time of the questionnaire. For the non-adherence analysis, inclusion criteria were: on ART at the time of the questionnaire, a non-missing value for age, and a non-missing value for at least one of two ART-adherence questions. ART non-adherence was defined as either an affirmative response to the question: “In the past 3 months, have you ever missed your HIV treatment for 2 or more days at a time?” or reporting one or more missed doses in response to the question: “In the last 2 weeks, how many doses of HIV treatment have you missed?”

For the virological non-suppression analysis, in addition to the criteria for the non-adherence analysis, individuals were required to: have a non-missing value for clinic-recorded viral load (the latest value at the time of questionnaire, using either the study recorded value or available linked clinic data); have a non-missing value for date of ART initiation; have started ART at least 6 months before the viral load measurement being used for analysis. Virological non-suppression was defined as viral load more than 50 copies per mL.

We summarised the prevalence of ART non-adherence and virological non-suppression according to demographic, socioeconomic, and social circumstance factors; groups were compared with the χ^2^ test or Cochran-Armitage test for trend for ordered categorical variables. Unadjusted and adjusted prevalence ratios for associations of socioeconomic and social circumstance factors with ART non-adherence and virological non-suppression, were generated using modified Poisson regression models.^24^ For multivariable models, each socioeconomic and social circumstance factor was considered in a separate model because of high co-linearity; associations were adjusted for demographic factors (gender/sexual orientation, ethnic origin, and age). We also assessed the association between ART non-adherence and virological non-suppression with modified Poisson regression, adjusted for demographic factors.

We did a subgroup analysis in white men who have sex with men to reduce confounding by demographic, ethnic, and cultural factors. We also did a sensitivity analysis in which a viral load of more than 200 copies per mL was defined as non-suppression, because low level viraemia might not be indicative of true virological failure.

### Longitudinal analysis

We did a longitudinal analysis to assess the associations of socioeconomic and social circumstance factors with risk of virological rebound. We included consenting ASTRA participants from the six centres for which linked clinic data were available. Baseline was defined as the date of questionnaire. Inclusion criteria were: on ART with viral load of 50 copies per mL or lower at baseline (latest value at the time of the questionnaire); started ART at least 6 months before the baseline viral load measurement; non-missing value for age; non-missing value for at least one ART-adherence question; and at least one viral load measurement subsequent to baseline. Individuals were followed up from baseline until virological rebound (defined as the first viral load >200 copies per mL) or the last available viral load (latest Oct 9, 2015). Follow up was not censored at ART interruption.

We assessed the unadjusted and adjusted associations of socioeconomic and social circumstance factors with subsequent virological rebound with Kaplan-Meier plots and Cox proportional hazards regression models. We used separate multivariate models for every socioeconomic and social circumstance factor, adjusted for demographic factors (gender/sexual orientation, ethnic origin, and age). Additionally, we assessed the association between ART non-adherence and viral load rebound with Cox proportional hazards regression, adjusted for demographic factors.

We did a subgroup analysis restricted to white men who have sex with men in order to reduce confounding. Two sensitivity analyses were done: virological rebound was defined as two consecutive viral load measurements more than 200 copies per mL to investigate an endpoint of sustained viral rebound; and those who were lost to follow-up (eligible for the longitudinal analysis but date of last measurement was more than 18 months before the clinic administrative censoring date) were regarded as having experienced virological rebound 6 months after the date of the last available viral load measurement, because lack of retention in care may be associated with poorer prognosis.

Complete-case analyses were done throughout because the proportion of participants with missing data did not exceed 4% for any variable used in the analysis.

We used SAS (version 9.3) for all statistical analyses.

### Role of the funding source

The funder of the study had no role in study design, data collection, data analysis, data interpretation, or writing of the report. The corresponding author had full access to all the data in the study and had final responsibility for the decision to submit for publication. FCL, CJS, and AS also had full access to the data.

## Results

Between Feb 1, 2011, and Dec 31, 2012, 5112 HIV-diagnosed men and women were invited to participate in the ASTRA study, of whom 4200 (82%) consented to take part. 3258 individuals completed the questionnaire (response rate 64% of the 5112 individuals approached). Of the 3258 people (69% men who have sex with men, 11% heterosexual men, and 20% women) who completed the questionnaire, 2771 (85%) reported being on ART at the time of the questionnaire. Of the remaining 487 (15%) people not on ART, 366 (11%) were ART-naive individuals, 65 (2%) had stopped ART, and 56 (2%) had missing ART information. Of the 2771 participants currently on ART, 58 (2%) had missing age, and nine (<1%) had not responded to either adherence question. This resulted in 2704 individuals being included (1867 men who have sex with men, 321 heterosexual men, and 516 women; table 1).

**Figure 1:**
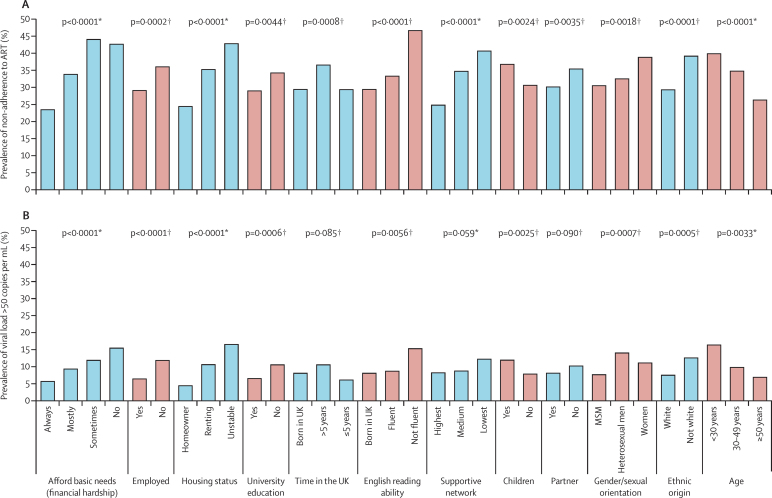
Prevalence of (A) antiretroviral therapy (ART) non-adherence and (B) virological non-suppression (viral load >50 copies per mL), by socioeconomic and demographic factors (A) Data taken from a cross-sectional analysis in 2704 respondents who were on ART at the time of the questionnaire. Self-reported ≥2 consecutive missed days of ART in the past 3 months or ≥1 missed dose in the last 2 weeks. (B) Data taken from a cross-sectional analysis in 2405 respondents who were on ART and had started ART >6 months before the viral load measurement. MSM=men who have sex with men. *Calculated with Cochran-Armitage test for trend. †Calculated with χ^2^ test.

Of the 2704 participants on ART, 873 (32%, 95% CI 31–34) reported ART non-adherence. Individuals with lower socioeconomic status by any measure (ie, increased financial hardship, non-employment, rented or unstable housing status, and non-university education) were more likely to report ART non-adherence (figure 1 and table 2). In terms of social circumstance factors, the prevalence of ART non-adherence was higher in individuals who had lived in the UK for more than 5 years but were not born in the UK, those who had non-fluent English reading ability, those who reported lower supportive network, those who had children, and those who did not have a current partner (figure 1 and table 2). After adjustment for demographic factors (gender/sexual orientation, ethnic origin, and age), all measures of poor socioeconomic status remained associated with an increased prevalence of ART non-adherence (table 2). Associations of non-adherence with non-fluent English, lower supportive network, having children, and no current partner also remained after adjustment for demographic factors, while the association with time in the UK was largely accounted for by the demographic factors (table 2). In a model that included only demographic factors, non-white ethnic origin (prevalence ratio [PR] 1·33 *vs* white, 95% CI 1·14–1·54) and younger age (PR 0·89 per 10 years older, 95% CI 0·84–0·95) were independently associated with non-adherence. However, we noted no independent association with gender/sexual orientation (PR 0·92 for heterosexual men and 0·99 for women *vs* men who have sex with men).

The virological non-suppression analysis included 2405 (89%) participants who had a recorded viral load and date of first ART initiation, and started ART more than 6 months before the viral load measurement. Of these, 219 (9%, 95% CI 8–10) had virological non-suppression (viral load >50 copies per mL; 79 [36%] with >500 copies per mL, 68 [31%] >1000 copies per mL, and 32 [15%] >10 000 copies per mL). As reported for ART non-adherence, for each of the four indicators of socioeconomic status, socioeconomic disadvantage was strongly associated with virological non-suppression (figure 1 and table 2). Additionally, individuals with non-fluent English reading ability and those who had children had an increased prevalence of virological non-suppression. There were weaker associations with non-suppression for individuals who were non-UK born and lived in the UK for more than 5 years, those who had lower supportive network, and those who had no current partner (figure 1 and table 2). Although socioeconomic disadvantage was strongly associated with non-suppression, the proportion of individuals with a viral load of more than 50 copies per mL was no more than 17% across all subgroups considered.

Table 2 also shows the adjusted associations of socioeconomic factors with virological non-suppression. The markers of lower socioeconomic status (financial hardship, non-employment, non-homeownership, and non-university education) all remained strongly associated with virological non-suppression after adjustment for demographic factors. We noted a marked trend between increasing prevalence of virological non-suppression and both increasing financial hardship and increasing housing instability. In terms of the additional social circumstance factors, having a lower supportive network and not having a current partner were associated with increased prevalence of virological non-suppression in the model adjusted for demographic factors. Living in the UK for less than 5 years was associated with lower prevalence of virological non-suppression compared with individuals born in the UK. The associations with low English fluency and having children were substantially attenuated. Because 76% of women had children compared with 7% of men who have sex with men, in unadjusted analyses the association between having children and higher prevalence of virological non-suppression could reflect an association with gender/sexual orientation.

In a model that included only demographic factors, younger age (PR 0·77 per 10 years older, 95% CI 0·66–0·88) was independently associated with virological non-suppression, and there was some evidence of associations of gender/sexual orientation (PR 1·45 for heterosexual men *vs* men who have sex with men, 95% CI 0·96–2·19 and 0·99 for women *vs* men who have sex with men, 95% CI 0·66–1·47) and ethnic origin (1·41 non-white *vs* white, 0·98–2·01) with non-suppression. Self-reported ART non-adherence was associated with 2·4 times higher prevalence of virological non-suppression (PR 2·37, 95% CI 1·84–3·07; p<0·0001), adjusted for demographic factors only.

Of 2405 participants included in the cross-sectional viral load analysis, 1740 (72%) had linked clinical data available and met the inclusion criteria for the longitudinal analysis (table 1). These individuals were followed up for 3818 person-years with a median of 2·4 years (IQR 2·0–2·7) of follow-up and a median of six (IQR 5–8) viral load measurements per person. During this period, eight (<1%) individuals died. During follow-up, 139 (8%) people had virological rebound, corresponding to a rate of 3·6 per 100 person-years (95% CI 3·0–4·2). By 12 and 24 months of follow-up, the Kaplan-Meier estimates of virological rebound were 3·9% (95% CI 3·0–4·8) and 7·0% (5·7–8·2), respectively.

**Figure 2:**
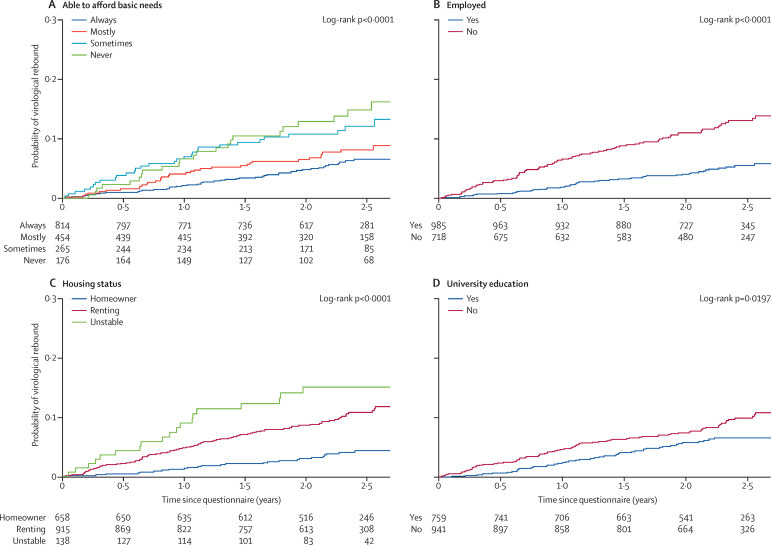
Kaplan-Meier plots of time until virological rebound (viral load >200 copies per mL) according to (A) ability to afford basic needs (financial hardship), (B) employment status, (C) housing status, and (D) university education Longitudinal analysis in 1740 respondents on ART with viral load <50 copies per mL at the time of the questionnaire. Individuals with missing values were excluded. Numbers provided indicate the number of individuals at risk.

In unadjusted Cox regression analysis, increased financial hardship, non-employment, and rented or unstable housing status were strongly predictive of increased risk of virological rebound (figure 2 and table 3). We noted a more modest association between non-university education and increased rebound risk (figure 2D and table 3). Additionally, having children and not having a partner were associated with a higher risk of rebound; data also suggested an association with lower supportive network. The pattern of associations remained, with some attenuation for some factors, after adjustment for demographic factors (table 3). In a model for viral load rebound containing only the demographic factors, there were independent associations of gender/sexual orientation (HR 2·00 for heterosexual men *vs* men who have sex with men, 95% CI 1·15–3·47; HR 1·45 for women *vs* men who have sex with men, 95% CI 0·87–2·41) and younger age (PR 0·74 per 10 years older, 95% CI 0·61–0·90) with virological rebound, but no evidence of association with ethnic origin (HR 1·32, 95% CI 0·83–2·09 for non-white *vs* white). Individuals who self-reported ART non-adherence at baseline had more than three times the rate of virological rebound compared with individuals who did not (HR 3·11, 95% CI 2·20–4·38; p<0·0001, adjusted for demographic factors only).

Among the subgroup of white men who have sex with men, the markers of lower socioeconomic status remained strongly associated with increased prevalence of ART non-adherence and virological non-suppression, and increased rates of virological rebound (appendix pp 1, 2). Results of all sensitivity analyses for cross-sectional and longitudinal analyses were broadly consistent with those of the main analyses (appendix pp 3, 4).

## Discussion

This is the first study to assess the effect of socioeconomic status on virological outcomes in people receiving treatment for HIV in the UK. In this setting of universal access to health care and high levels of treatment success, all four markers of lower socioeconomic status considered (financial hardship, non-employment, rented or unstable housing status, and non-university education) were strongly associated with ART non-adherence and virological non-suppression on ART. Furthermore, each of the four markers of lower socioeconomic status was predictive of subsequent virological rebound in people with viral suppression at baseline. These results provide evidence of the importance of current socioeconomic disadvantage in determining virological outcomes of ART. The adverse implications of poorer socioeconomic status clearly go beyond inability to pay for treatment and health care, and operate strongly even in people engaged with clinical care.

In previous European studies^11^, ^12^, ^13^, ^14^ of socioeconomic status and ART outcomes in which education level was used as the sole indicator of socioeconomic status, lower education level was associated with virological non-suppression in two of three studies. Two additional European studies considered employment status and noted that unemployment was associated with twice the adjusted risk of virological failure^15^ and that viral load of more than 50 copies per mL was associated with twice the unadjusted odds of developing inability to work among those able to work when starting ART.^25^ In terms of mortality risk, in a French study of individuals starting ART, social vulnerability (combining education, employment, and housing status) was associated with 20% increased mortality risk after adjustment for behavioural and biomedical factors.^26^ The results of this present analysis add to existing findings showing strong associations between current markers of poverty and hardship and viral load response to ART in people with HIV in the UK.

Adherence to treatment is the strongest determinant of virological response to ART.^16^ The strong association between socioeconomic factors and ART non-adherence, and between non-adherence and virological outcomes, suggest that associations between low socioeconomic status and virological non-suppression are probably mediated mainly through ART non-adherence. It is important to appreciate the apparent substantial effect of socioeconomic factors on non-adherence, even in the current era of simpler and more tolerable drugs, with most participants on once a day regimens. There are a number of reasons why people with greater levels of social or financial disadvantage might have greater difficulties maintaining treatment adherence, including competing responsibilities and stress, unsettled living circumstances, food insecurity (particularly when ART regimen requires food),^27^ increased prevalence of mental health problems,^18^ stigma and low self-esteem, or less knowledge about the importance of adherence.^28^ It is also conceivable that part of the effect of socioeconomic status on virological outcomes is independent of non-adherence, for example related to factors such as late diagnosis,^29^ low CD4 count or AIDS at ART initiation,^30^ differences in experiences or quality of health care, and pharmacokinetics through absence of sufficient food.^31^

The results of this study have practical implications to guide the identification of individuals on ART who are at higher risk of ART non-adherence and poorer treatment outcomes. Individuals with difficult socioeconomic circumstances might benefit from specific support with ART adherence such as prescription of less complex regimens,^32^ or interventions such as peer support.^33^ Moreover, the results show that the success of treatment cannot be separated from the social context in which it occurs. They emphasise the importance of a holistic approach to HIV care, with awareness that difficulties in individuals' circumstances affect treatment success, and good links to social care services that can support individuals in addressing difficulties with finance and benefits, housing, and employment issues. However, our findings also raise the agenda of socioeconomic inequalities in health in a wider context, adding to existing evidence of the adverse effect of poverty and social disadvantage on health outcomes.^34^ Socioeconomic factors are often not incorporated in clinical research studies of HIV; however, our results show that such factors are likely to be profound determinants of HIV outcomes. As such, there is a need for systematic collection of socioeconomic factors in HIV clinical care and research.

There are some limitations to this study. The ASTRA questionnaire study response rate was 64%: non-responders might differ from responders with regard to socioeconomic factors and association with virological outcomes. Our sample had a lower proportion of black African individuals, a lower proportion of individuals who acquired HIV through heterosexual sex, and a greater proportion of men who have sex with men than among people living with HIV in the UK generally.^35^ We did not account for whether participants were on first-line or subsequent ART regimens, and the specific regimen used. Our measures of socioeconomic status and adherence to ART were collected at one timepoint only and by self-report. We did not include 65 individuals who had previously been on ART but were not on ART at the time of the questionnaire; when this group were included in cross-sectional analyses as non-adherent, the prevalence of non-adherence and non-suppression was slightly higher than seen in the main analysis (34% *vs* 32%, and 11% *vs* 9% respectively), but socioeconomic associations were unchanged (data not shown). In the cross-sectional analysis, only association can be studied; it is not possible to rule out the presence of reverse causality for some factors. However, all findings are reinforced in the longitudinal analysis, which is unlikely to suffer from this bias. Longitudinal time-to-rebound analyses are potentially subject to bias if frequency of viral load monitoring differs according to explanatory variables; however, the median number of viral load measurements during follow-up was very similar across socioeconomic subgroups (data not shown).

In summary, even in a European setting with free access to HIV treatment and overall high rates of treatment success, socioeconomic disadvantage substantially affects HIV treatment outcomes. Emphasis should be placed on supporting adherence of people in these higher risk groups. Socioeconomic factors should be taken into account when designing clinical management strategies including linkage to the relevant social care agencies. Further research is needed on specific interventions that reduce socioeconomic inequalities in HIV-outcomes.

## Figures and Tables

**Table 1:** Participants' characteristics

	Cross-sectional analysis*; participants included in non-adherence analysis (N=2704)†	**Longitudinal analysis**‡**; participants included in viral load rebound analysis (N=1740)**†
**Gender/sexual orientation**		
Men who have sex with men	1867 (69%)	1267 (73%)
Heterosexual men	321 (12%)	171 (10%)
Women	516 (19%)	302 (17%)
**Risk group**		
Sex between men	1748 (65%)	1195 (69%)
Heterosexual sex	536 (20%)	314 (18%)
Injecting drug use	46 (2%)	25 (1%)
Other	353 (13%)	197 (11%)
Missing	21 (1%)	9 (1%)
**Ethnic origin**		
White	1875 (69%)	1259 (72%)
Black African	507 (19%)	281 (16%)
Black other	89 (3%)	52 (3%)
Other	184 (7%)	113 (6%)
Missing	49 (2%)	35 (2%)
**Age**		
Median (IQR)	46 (40–52)	46 (41–52)
**Afford basic needs (financial hardship)**§		
Always	1170 (43%)	814 (47%)
Mostly	701 (26%)	454 (26%)
Sometimes	464 (17%)	265 (15%)
No	326 (12%)	176 (10%)
Missing	43 (2%)	31 (2%)
**Employment**		
Employed	1479 (55%)	985 (57%)
Unemployed	483 (18%)	286 (16%)
Sick or disabled	375 (14%)	224 (13%)
Retired	180 (7%)	129 (7%)
Other	127 (5%)	79 (5%)
Missing	49 (2%)	37 (2%)
**Housing**		
Homeowner	914 (35%)	658 (38%)
Renting from council	840 (31%)	522 (30%)
Renting privately	609 (23%)	393 (23%)
Temporary accommodation or homeless	70 (3%)	35 (2%)
Staying with family	191 (7%)	97 (6%)
Other	10 (<1%)	6 (<1%)
Missing	40 (1%)	29 (2%)
**Education (highest level)**		
University degree or higher	1094 (40%)	759 (44%)
A-level or equivalent	536 (20%)	338 (19%)
O-levels or equivalent	601 (22%)	364 (21%)
Other	108 (4%)	70 (4%)
None	302 (11%)	169 (10%)
Missing	63 (2%)	40 (2%)
**Time in UK**		
Born in UK	1511 (56%)	983 (56%)
>5 years	991 (37%)	635 (36%)
≤5 years	116 (4%)	68 (4%)
Missing	86 (3%)	54 (3%)
**English reading ability**		
Born in UK	1511 (56%)	983 (56%)
Fluent	912 (34%)	595 (34%)
Not fluent	208 (8%)	114 (7%)
Missing	73 (3%)	48 (3%)
**Supportive network**		
Most support	878 (32%)	562 (32%)
Medium support	1414 (52%)	930 (53%)
Least support	377 (14%)	227 (13%)
Missing	35 (1%)	21 (1%)
**Children**		
Yes	733 (27%)	426 (24%)
No	1954 (72%)	1305 (75%)
Missing	17 (1%)	9 (1%)
**Partner**		
Yes	1530 (57%)	997 (57%)
No	1158 (43%)	731 (42%)
Missing	16 (1%)	12 (1%)
**Time since HIV diagnosis**		
<2 years	180 (7%)	64 (4%)
2–5 years	361 (13%)	222 (13%)
5–15 years	1345 (50%)	926 (53%)
>15 years	755 (28%)	528 (30%)
Missing	63 (2%)	0
**Number of times taking ART per day**		
1	2159 (80%)	1419 (81%)
≥2	513 (19%)	309 (18%)
Missing	32 (1%)	21 (1%)
**≥2 consecutive missed days of ART in the past 3 months**		
No or unknown	2236 (83%)	1461 (84%)
Yes	464 (17%)	277 (16%)
Missing	4 (<1%)	2 (<1%)
**≥1 missed dose in the past 2 weeks**		
No or unknown	2022 (75%)	1289 (74%)
Yes	676 (25%)	447 (26%)
Missing	6 (<1%)	4 (<1%)
**Non-adherent to ART**¶		
No or unknown	1831 (68%)	1174 (67%)
Yes	873 (32%)	566 (33%)
**Time on ART (years)**‖		
Median (IQR)	6·9 (2·8–12·4)	7·7 (3·7–12·9)
**CD4 count (cells/mm^3^)**‖		
Median (IQR)	546 (393–732)	590 (442–780)
**Viral load at the time of the questionnaire**		
≤50 copies per mL	2347 (87%)	1740 (100%)
>50 copies per mL	341 (13%)**	0
Missing	16 (1%)	0

Data are n (%) unless stated otherwise. ART=antiretroviral therapy.

* All participants who self-reported being on ART at the time of the questionnaire and had recorded age and non-adherence information. Of these, 2405 participants had a recorded viral load and date of ART initiation, and additionally started ART >6 months before the viral load measurement and were included in viral load non-suppression analysis.

† Some column percentages do not add to 1 because of rounding.

‡ All participants had linked clinical data, recorded age and non-adherence information, were on ART, had viral load ≤50 copies per mL at the time of the questionnaire, started ART >6 months before the baseline viral load measurement, and had ≥1 subsequent viral load measurement.

§ Defined as having money for basic needs.

¶ Self-reported ≥2 consecutive missed days of ART in the past 3 months or ≥1 missed dose in the past 2 weeks.

‖ Cross-sectional N=99 and longitudinal N=0 with missing time on ART, cross-sectional N=17 and longitudinal N=2 with missing CD4 count.

** Among 2405 participants that had additionally started ART >6 months before completion of the questionnaire and were included in viral load non-suppression analysis, 219 (9%) had viral load >50 copies per mL.

**Table 2:** Associations of socioeconomic factors with antiretroviral non-adherence*and virological non-suppression†

	**ART non-adherence**‡				**Viral load non-suppression**§			
	Unadjusted		Adjusted for demographic factors¶		Unadjusted		Adjusted for demographic factors¶	
	PR (95% CI)	p value‖	aPR (95% CI)	p value‖	PR (95% CI)	p value‖	aPR (95% CI)	p value‖
**Enough money for basic needs? (financial hardship)**								
Always	1	<0·0001**	1	<0·0001**	1	<0·0001**	1	<0·0001
Mostly	1·44 (1·24–1·66)		1·42 (1·22–1·64)		1·63 (1·15–2·30)		1·56 (1·11–2·21)	
Sometimes	1·88 (1·62–2·17)		1·81 (1·56–2·11)		2·06 (1·44–2·95)		1·84 (1·26–2·68)	
No	1·82 (1·55–2·14)		1·74 (1·46–2·06)		2·68 (1·87–3·86)		2·35 (1·60–3·43)	
**Employed**								
Yes	1	0·0002	1	<0·0001	1	<0·0001	1	<0·0001
No	1·24 (1·11–1·38)		1·29 (1·15–1·45)		1·85 (1·42–2·41)		1·96 (1·49–2·58)	
**Housing status**								
Homeowner	1	<0·0001**	1	<·0001**	1	<0·0001**	1	<0·0001
Renting	1·44 (1·27–1·65)		1·34 (1·17–1·54)		2·39 (1·69–3·39)		2·09 (1·46–2·98)	
Unstable	1·76 (1·47–2·10)		1·58 (1·31–1·91)		3·70 (2·42–5·67)		2·96 (1·90–4·59)	
**University education**								
Yes	1	0·0041	1	0·0028	1	0·0004	1	0·0003
No	1·18 (1·05–1·33)		1·19 (1·06–1·34)		1·63 (1·23–2·16)		1·63 (1·23–2·16)	
**Time in the UK**								
Born in the UK	1	0·0010	1	0·086	1	0·083	1	0·044
>5 years	1·24 (1·11–1·39)		1·07 (0·93–1·24)		1·30 (1·00–1·69)		0·89 (0·65–1·22)	
≤5 years	1·00 (0·75–1·34)		0·80 (0·59–1·08)		0·75 (0·34–1·67)		0·45 (0·20–1·02)	
**English reading ability**								
Born in UK	1	<0·0001	1	0·0048	1	0·036	1	0·066
Fluent	1·13 (1·00–1·28)		1·00 (0·87–1·16)		1·09 (0·82–1·45)		0·77 (0·55–1·07)	
Not fluent	1·59 (1·35–1·88)		1·37 (1·12–1·67)		1·89 (1·29–2·78)		1·19 (0·74–1·93)	
**Supportive network**								
Most	1	<0·0001**	1	<0·0001**	1	0·071**	1	0·031
Medium	1·39 (1·22–1·60)		1·40 (1·22–1·60)		1·07 (0·80–1·44)		1·12 (0·83–1·51)	
Least	1·63 (1·38–1·93)		1·65 (1·39–1·95)		1·49 (1·03–2·15)		1·59 (1·10–2·30)	
**Children**								
Yes	1	0·0030	1	0·022	1	0·0053	1	0·29
No	0·83 (0·74–0·94)		0·83 (0·70–0·97)		0·67 (0·51–0·87)		0·80 (0·53–1·21)	
**Partner**								
Yes	1	0·0037	1	0·0014	1	0·094	1	0·026
No	1·18 (1·06–1·31)		1·20 (1·07–1·34)		1·25 (0·97–1·61)		1·35 (1·04–1·75)	

Each socioeconomic factor considered in a separate model for all results; individuals with missing values for explanatory variables were excluded. ART=antiretroviral therapy. PR=prevalence ratio. aPR=adjusted prevalence ratio.

* Self-reported ≥2 consecutive missed days of ART in the past 3 months or ≥1 missed dose in the last 2 weeks.

† Viral load >50 copies per mL at the time of the questionnaire.

‡ Cross-sectional analysis in 2704 respondents who were on ART at the time of the questionnaire.

§ Cross-sectional analysis among 2405 respondents who were on ART and had started ART >6 months before the viral load measurement.

¶ Gender/sexual orientation–ethnic origin–and age.

‖ Calculated with χ^2^ test.

** Test for trend.

**Table 3:** Associations of socioeconomic factors with virological rebound (viral load >200 copies per mL)*

	**N**	**Rate per 100 person-years**	**Unadjusted**		**Adjusted for demographic factors**†	
			HR (95% CI)	p value‡	aHR (95% CI)	p value§
**Enough money for basic needs? (financial hardship)**						
Always	814	2·49	1	<0·0001§	1	0·0005§
Mostly	454	3·64	1·47 (0·95–2·27)		1·34 (0·86–2·09)	
Sometimes	265	5·60	2·25 (1·43–3·55)		1·86 (1·15–3·01)	
No	176	6·95	2·78 (1·71–4·53)		2·34 (1·39–3·92)	
**Employed**						
Yes	985	2·26	1	<0·0001	1	<0·0001
No	718	5·78	2·56 (1·81–3·62)		2·95 (2·05–4·25)	
**Housing status**						
Homeowner	658	1·70	1	<0·0001§	1	<0·0001§
Renting	915	4·76	2·80 (1·81–4·32)		2·40 (1·52–3·79)	
Unstable/other	138	6·95	4·11 (2·27–7·42)		3·30 (1·77–6·13)	
**University education**						
Yes	759	2·79	1	0·021	1	0·014
No	941	4·24	1·52 (1·07–2·17)		1·57 (1·10–2·26)	
**Time in the UK**						
Born in UK	983	3·09	1	0·11	1	0·51
In UK >5 years	635	4·47	1·44 (1·02–2·04)		0·95 (0·61–1·48)	
In UK ≤5 years	68	2·96	0·95 (0·35–2·59)		0·54 (0·19–1·54)	
**English reading ability**						
Born in UK	983	3·09	1	0·14	1	0·81
Fluent	595	4·35	1·40 (0·98–2·00)		0·92 (0·59–1·42)	
Not fluent	114	4·43	1·43 (0·74–2·78)		0·78 (0·37–1·66)	
**Supportive network**						
Most support	562	3·07	1	0·070§	1	0·044§
Medium support	930	3·68	1·20 (0·81–1·77)		1·20 (0·81–1·87)	
Least support	227	5·04	1·63 (0·98–2·72)		1·76 (1·05–2·94)	
**Children**						
Yes	426	6·03	1	<0·0001	1	0·014
No	1305	2·91	0·49 (0·34–0·68)		0·53 (0·32–0·88)	
**Partner**						
Yes	997	2·96	1	0·0081	1	0·0021
No	731	4·65	1·57 (1·12–2·19)		1·71 (1·21–2·40)	

Every socioeconomic factor was considered in a separate model for all results; individuals with missing values for explanatory variables were excluded. HR=hazard ratio. aHR=adjusted hazard ratio.

* Longitudinal analysis in 1740 respondents on ART with viral load <50 copies per mL at the time of the questionnaire.

† Gender/sexual orientation, ethnic origin, and age.

‡ Calculated with χ^2^ test.

§ Test for trend.
