# Supplementary material for: Socioeconomic status and treatment outcomes for individuals with HIV on antiretroviral treatment in the UK: cross-sectional and longitudinal analyses
Source: Lancet Public Health. 2016 Nov;1(1):e26–36. doi: 10.1016/S2468-2667(16)30002-0 (PMC5341147; doi:10.1016/S2468-2667(16)30002-0)
Supplement: Supplementary appendix [file mmc1.pdf]

THE LANCET Public Health

Supplementary appendix

This appendix formed part of the original submission and has been peer reviewed. We post it as supplied by the authors.

Supplement to: Burch LS, Smith CJ, Anderson J, et al, for the Antiretrovirals, Sexual Transmission Risk and Attitudes (ASTRA) Study Group. Socioeconomic status and treatment outcomes for individuals with HIV on antiretroviral treatment in the UK: cross-sectional and longitudinal analyses. *Lancet Public Health* 2016; published online Oct 12. http://dx.doi.org/10.1016/S2468-2667(16)30002-0.

Supplementary table 1: Cross-sectional analyses restricted to the subgroup of white MSM only (N=1665 in non-adherence analysis and N=1498 in non-suppression analysis)

**Factors** [a]			**ART non-adherence** [b]							**Viral load non-suppression** [c]					
			Unadjusted			Adjusted for age				Unadjusted			Adjusted for age		
		N	PR	95% CI	P-value [d]	PR	95% CI	P-value [d]	N	PR	95% CI	P-value [d]	aPR	95% CI	P-value [d]
Enough money for basic needs? (Financial hardship)	Always	899	1		<.0001 [e]	1		<.0001 [e]	813	1		0.0041 [e]	1		0.0055 [e]
	Mostly	450	1.48	(1.24, 1.76)		1.47	(1.23, 1.75)		407	1.48	(0.96, 2.26)		1.45	(0.95, 2.23)	
	Sometimes	195	1.88	(1.54, 2.29)		1.84	(1.51, 2.25)		172	2.06	(1.25, 3.38)		2.00	(1.22, 3.28)	
	No	120	1.47	(1.12, 1.94)		1.45	(1.10, 1.90)		105	2.02	(1.11, 3.69)		1.95	(1.07, 3.56)	
Employed	Yes	999	1		0.0070	1		0.0003	887	1		0.0021	1		0.0002
	No	658	1.23	(1.06, 1.43)		1.34	(1.15, 1.56)		603	1.79	(1.25, 2.57)		2.12	(1.47, 3.08)	
Housing status	Homeowner	743	1		0.0002 [e]	1		0.0018 [e]	677	1		<.0001 [e]	1		0.0001 [e]
	Renting	814	1.27	(1.09, 1.49)		1.23	(1.04, 1.45)		731	2.13	(1.41, 3.23)		1.99	(1.32, 3.01)	
	Unstable/ other	106	1.57	(1.21, 2.05)		1.50	(1.14, 1.97)		88	3.33	(1.81, 6.15)		3.06	(1.67, 5.62)	
University education	Yes	722	1		0.0040	1		0.0027	650	1		0.0008	1		0.0006
	No	938	1.25	(1.07, 1.46)		1.26	(1.08, 1.47)		844	1.90	(1.28, 2.83)		1.93	(1.30, 2.88)	

[a] Each socio-economic factor considered in a separate model for all results, individuals with missing values for socio-economic factors were excluded; [b] self-reported ART non-adherence: ≥1 missed dose in the past 3 months for ≥2 consecutive days or ≥1 missed dose in the last 2 weeks; [c] viral load >50 copies/mL at the time of the questionnaire; [d] Chi square test; [e] test for trend; PR=Prevalence Ratio; aPR=adjusted Prevalence Ratio.

Supplementary table 2: Longitudinal analysis restricted to the subgroup of white MSM only (N=1131)

Factors [a]		N	Rate [b]	Unadjusted			Adjusted for age		
				HR	95% CI	P-value [c]	aHR	95% CI	P-value [c]
Enough money for basic needs? (Financial hardship)	Always	651	2.41	1		0.24 [d]	1		0.32 [d]
	Mostly	296	2.73	1.10	(0.63, 1.94)		1.08	(0.62, 1.90)	
	Sometimes	114	4.83	1.93	(1.00, 3.69)		1.86	(0.97, 3.56)	
	No	69	2.53	1.03	(0.37, 2.90)		0.96	(0.34, 2.71)	
Employed	Yes	703	2.05	1		0.011	1		0.0004
	No	424	3.91	1.84	(1.15, 2.93)		2.38	(1.47, 3.86)	
Housing status	Homeowner	527	1.51	1		0.023 [d]	1		0.071 [d]
	Renting	545	4.01	2.34	(1.40, 3.92)		2.11	(1.25, 3.57)	
	Unstable/ other	57	1.56	0.88	(0.21, 3.76)		0.80	(0.19, 3.44)	
University education	Yes	525	1.84	1		0.017	1		0.012
	No	604	3.53	1.83	(1.11, 3.01)		1.89	(1.15, 3.10)	

[a] Each socio-economic factor considered in a separate model for all results, individuals with missing values for socio-economic factors were excluded; [b] per 100 person-years; [c] Chi square test; [d] test for trend; HR= Hazard Ratio; aHR= adjusted Hazard Ratio.

Supplementary table 3: Cross-sectional sensitivity analysis - virological non-suppression defined as viral load >200 copies/mL (N= 2405)

Factors [a]			Viral load non-suppression					
			Unadjusted			Adjusted for demographic factors [b]		
		N	PR	95% CI	P-value [c]	aPR	95% CI	P-value [c]
Enough money for basic needs? (Financial hardship)	Always	1038	1		<·0001 [d]	1		0·0001 [d]
	Mostly	627	1.34	(0.80, 2.24)		1.30	(0.78, 2.19)	
	Sometimes	412	2.52	(1.55, 4.09)		2.34	(1.39, 3.95)	
	No	290	3.12	(1.89, 5.14)		2.92	(1.71, 4.98)	
Employed	Yes	1302	1		<·0001	1		<·0001
	No	1049	2.35	(1.60, 3.45)		2.59	(1.73, 3.88)	
Housing status	Homeowner	852	1		<·0001 [d]	1		0·0002 [d]
	Renting	1286	3.08	(1.84, 5.16)		2.68	(1.59, 4.54)	
	Unstable/ other	230	3.92	(2.05, 7.49)		3.07	(1.56, 6.04)	
University education	Yes	977	1		0·0003	1		0·0003
	No	1370	2.00	(1.33, 3.00)		2.02	(1.34, 3.03)	

[a] Each socio-economic factor considered in a separate model for all results, individuals with missing values for socio-economic factors were excluded; [b] gender/ sexual orientation, ethnicity, age; [c] Chi square test; [d] test for trend; PR=Prevalence Ratio; aPR=adjusted Prevalence Ratio

Supplementary table 4 – Longitudinal sensitivity analyses

Factors [a]		N	Rate [c]	Unadjusted			Adjusted for demographics [b]		
				HR	95% CI	P-value [d]	aHR	95% CI	P-value [d]
**Sensitivity analysis 1: virological rebound defined as initial viral load ≤50 copies/mL and subsequently two consecutive viral load measurements >200 copies/mL (N=1717)**									
Enough money for basic needs? (Financial hardship)	Always	810	1.17	1		<.0001 [e]	1		0.0010 [e]
	Mostly	448	2.30	1.98	(1.10, 3.54)		1.80	(1.00, 3.25)	
	Sometimes	258	1.92	1.64	(0.79, 3.38)		1.29	(0.60, 2.74)	
	No	170	5.21	4.38	(2.37, 8.10)		3.57	(1.86, 6.87)	
Employed	Yes	975	1.12	1		<.0001	1		<.0001
	No	705	3.20	2.86	(1.77, 4.63)		3.25	(1.98, 5.33)	
Housing status	Homeowner	654	0.78	1		<.0001 [e]	1		0.0033 [e]
	Renting	899	2.71	3.50	(1.87, 6.54)		2.79	(1.46, 5.32)	
	Unstable/ other	135	3.19	4.17	(1.76, 9.90)		3.00	(1.23, 7.34)	
University education	Yes	749	1.11	1		0.0016	1		0.0015
	No	928	2.59	2.33	(1.38, 3.92)		2.33	(1.38, 3.94)	
**Sensitivity analysis 2: lost to follow-up considered virological rebound (viral load ≤50 and 1 subsequent viral load >200)[f] (N=1740)**									
Enough money for basic needs? (Financial hardship)	Always	814	3.97	1		<.0001 [e]	1		<.0001 [e]
	Mostly	454	5.79	1.46	(1.03, 2.06)		1.35	(0.95, 1.91)	
	Sometimes	265	8.36	2.11	(1.46, 3.04)		1.76	(1.20, 2.59)	
	No	176	11.89	3.02	(2.08, 4.39)		2.54	(1.71, 3.78)	
Employed	Yes	985	3.98	1		<.0001	1		<.0001
	No	718	8.66	2.18	(1.67, 2.85)		2.45	(1.85, 3.24)	
Housing status	Homeowner	658	2.60	1		<.0001 [e]	1		<.0001 [e]
	Renting	915	7.61	2.92	(2.06, 4.14)		2.49	(1.73, 3.58)	
	Unstable/ other	138	11.39	4.38	(2.75, 6.97)		3.53	(2.17, 5.74)	
University education	Yes	759	4.87	1		0.037	1		0.031
	No	941	6.50	1.34	(1.02, 1.76)		1.36	(1.03, 1.79)	

[a] Each socio-economic factor considered in a separate model for all results, individuals with missing values for socio-economic factors were excluded; [b] gender/ sexual orientation, ethnicity, age; [c] per 100 person-years; [d] Chi square test; [e] test for trend; [f] lost to follow-up defined as consented longitudinal linkage and ≥1 viral load measurement after questionnaire date but latest follow-up viral load over eighteen months before administrative censoring date, date of rebound for these individuals is the date of but latest follow-up viral load plus six months; HR= Hazard Ratio; aHR= adjusted Hazard Ratio.

ASTRA clinic teams

Royal Free Hospital: Alison Rodger; Margaret Johnson; Jeff McDonnell; Aderonke Adebiyi

Mortimer Market Centre: Richard Gilson; Simon Edwards; Lewis Haddow; Simon Gilson; Christina Broussard; Robert Pralat; Sonali Wayal

Brighton and Sussex University Hospital: Martin Fisher; Nicky Perry; Alex Pollard; Serge Fedele; Louise Kerr; Lisa Heald; Wendy Hadley; Kerry Hobbs; Julia Williams; Elaney Youssef; Celia Richardson; Sean Groth

North Manchester General Hospital: Ed Wilkins; Yvonne Clowes; Jennifer Cullie; Cynthia Murphy; Christina Martin; Valerie George; Andrew Thompson

Homerton University Hospital: Jane Anderson; Sifiso Mguni; Damilola Awosika; Rosalind Scourse

East Sussex Sexual Health Clinic: Kazeem Aderogba; Caron Osborne; Sue Cross; Jacqueline Whinney; Martin Jones

Newham University Hospital: Rebecca O'Connell; Cheryl Tawana

Whipps Cross University Hospital: Monica Lascar; Zandile Maseko; Gemma Townsend; Vera Theodore; Jas Sagoo

ASTRA core team: Fiona Lampe; Alison Rodger; Andrew Speakman; Andrew Phillips

ASTRA data management: Andrew Speakman; Marina Daskalopoulou; Fiona Lampe

ASTRA advisory group: Lorraine Sherr; Simon Collins; Jonathan Elford ; Alec Miners; Anne Johnson; Graham Hart; Anna-Maria Geretti; Bill Burman

CAPRA grant Advisory Board: Nick Partridge; Kay Orton; Anthony Nardone; Ann Sullivan
